# Treatment methods and recurrence rates in primary spontaneous pneumothorax: a 10-year retrospective study

**DOI:** 10.4314/ahs.v26i2.13

**Published:** 2026-06

**Authors:** Miktat Arif Haberal, Mehmet Ali Çolak, Hakan Demirci, Özlem Şengören Dikiş, Melih Yüksel

**Affiliations:** 1 University of Health Sciences, Bursa Yuksek Ihtisas Training and Research Hospital, Department of Thoracic Surgery, Bursa, Turkey; 2 University of Health Sciences, Bursa Yuksek Ihtisas Training and Research Hospital, Department of Family Medicine, Bursa, Turkey; 3 Muğla Sıtkı Koçman University School of Medicine, Department of Pulmonary Medicine, Muğla, Türkiye; 4 University of Health Sciences, Bursa Yuksek Ihtisas Training and Research Hospital, Department of Emergency Medicine, Bursa, Turkey

**Keywords:** Pleural Diseases, Primary Spontaneous Pneumothorax, Recurrence, Risk Factors, Smoking, Thoracoscopic Surgery Video-Assisted, Treatment Outcome

## Abstract

**Aim:**

Primary spontaneous pneumothorax (PSP) predominantly affects young individuals and presents with variable treatment outcomes and considerable recurrence rates. Understanding the relationship between treatment approach and recurrence is essential for guiding clinical decision-making. The aim of this study was to evaluate demographic characteristics, treatment modalities, and recurrence rates in patients with PSP.

**Methods:**

This single-center, retrospective study included patients diagnosed with PSP between January 2014 and December 2023. Recurrence rates following treatment were analyzed.

**Results:**

A total of 314 patients (median age 28 years; 93.0% male) were included. Tube thoracostomy was the initial treatment in 250 patients (79.6%). Recurrence occurred in 78 patients (24.8%), with higher rates following tube thoracostomy (28.0%) compared to oxygen therapy (17.8%). No recurrence was observed after initial video-assisted thoracoscopic surgery (VATS). In recurrent cases, 44.9% underwent VATS, 25.6% tube thoracostomy, and 29.5% oxygen therapy. Treatment choice and smoking status showed significant associations (p<0.001 for the first episode and p=0.007 for the second episode). Notably, all patients who underwent VATS were smokers.

**Conclusions:**

VATS performed during the first pneumothorax episode in selected patients prevents recurrence. The feasibility of VATS as the first treatment option in selected patients should be further investigated in larger series.

## Introduction

Pneumothorax is defined as the accumulation of free air between the visceral and parietal pleura^1^. It is classified into spontaneous, traumatic, and iatrogenic types^2^. Spontaneous pneumothorax (SP) is further divided into primary spontaneous pneumothorax (PSP), which occurs without underlying lung disease, and secondary spontaneous pneumothorax (SSP), which occurs in the presence of lung pathology^3^. The most commonly accepted mechanism of PSP is the rupture of apical bullae and blebs^1^,^4^.

PSP is frequently observed in young, tall, and thin individuals with low BMI, and has a strong association with smoking^5^,^6^. Atmospheric pressure variations have also been suggested as a contributing factor in the development of SP^7^. The incidence of PSP is reported as 8–28 per 100,000 per year in men and 1.2–6 per 100,000 per year in women, with men being affected approximately six times more frequently^8^.

Most patients with PSP present with chest pain and dyspnea^1^. The primary goals of treatment are to achieve lung re-expansion and prevent recurrence^3^. Posterior-anterior and lateral chest radiographs are the recommended first-line imaging modalities, whereas computed tomography is reserved for diagnostic uncertainty^3^. Treatment options include observation, oxygen therapy, needle aspiration, catheter drainage, tube thoracostomy, video-assisted thoracoscopic surgery (VATS), and thoracotomy^3^,^5^. The annual recurrence rate in PSP patients ranges from 13% to 49%, depending on the treatment method used^9^. Recent studies have reported that VATS provides successful, efficient, and safe outcomes, with significantly lower recurrence rates compared with chest tube insertion^9^-^12^.

In light of these findings, evaluating recurrence in relation to treatment modalities remains clinically important. We hypothesized that VATS, when performed during the first episode in selected PSP patients, would be associated with significantly lower recurrence rates compared with tube thoracostomy or oxygen therapy.

## Methods

This single-center, retrospective study was conducted at the Thoracic Surgery Clinic of Bursa Yuksek Ihtisas Training and Research Hospital between January 2014 and December 2023. A total of 314 PSP patients (292 male, 22 female) were included. The age range of the patients was 18-39 years (median age 28). All patients were followed up and treated in our clinic. Patients under 18 and over 39 years of age were excluded from the study as they did not meet the PSP criteria. Demographic characteristics (age, sex), pneumothorax location, smoking history, treatment methods, and recurrence rates were retrospectively analyzed. Data were obtained from patient files and the Radiology department. Ethical approval for the study (2024-TBEK 2024/11-04) was granted by theSBÜ Bursa Yuksek Ihtisas Training and Research Hospital Ethics Committee.

The size of the pneumothorax was calculated using the methods recommended by the British and American Thoracic Societies (3,13). A small pneumothorax was defined as a distance of ≤3 cm between the lung apex and the chest wall or ≤2 cm at the hilum level. A large pneumothorax was defined as a distance of >3 cm between the lung apex and the chest wall or >2 cm at the hilum level. Small pneumothoraces were managed with oxygen therapy and observation, consistent with guideline recommendations. Patients whose symptoms improved and whose pneumothorax size decreased were discharged. Large pneumothoraces were treated with tube thoracostomy, and prophylactic antibiotic therapy was initiated (3,13). The chest drain was clamped for 12-24 hours if air leakage ceased and the lung re-expanded. The drain was removed if no pneumothorax was observed on chest X-ray and no air leakage was detected upon unclamping. Follow-up visits were scheduled at 10 days, 1 month, 3 months, and 1 year after discharge. This follow-up schedule reflects our institutional practice.

A formal sample size calculation was not performed. The condition investigated in this study represents a relatively rare clinical entity, and therefore achieving a large number of eligible cases prospectively is not feasible. However, based on the highest reported prevalence in the literature (8), theoretical sample size estimation was conducted using the formula: n = Z^2^ × P × Q / d^2^, where P = 0.00028, Q = 1 − P = 0.99972, Z = 1.96 and d = 0.01, yielding a minimum of 11 required participants. Accordingly, the study aimed to include all accessible cases during the defined study period, and the final sample surpassed the estimated requirement.

## Statistical Analysis

Descriptive statistics were expressed as mean ± standard deviation, median with range, and/or interquartile range (IQR) for numerical variables, while categorical variables were expressed as counts and percentages. The Kolmogorov-Smirnov test was used to assess the normality of the data distribution. The homogeneity of variances was tested using Levene's test. Fisher's exact test was used to analyze the relationship between categorical variables. A p-value of <0.05 was considered statistically significant, and all statistical tests were two-sided. Statistical analyses were performed using IBM SPSS Statistics for Windows, Version 21.0 (IBM Corp., Armonk, NY, USA). Results were reported with a 95% confidence interval.

## Results

### Baseline Characteristics

A total of 314 patients were included in the study. The median age of the patients was 28 years (IQR: 24–35), and 292 (93.0%) were male. A history of smoking was present in 232 patients (73.9%).

### Treatment Modalities during the First Episode

During the first pneumothorax episode, tube thoracostomy was performed in 250 patients (79.6%), while VATS was performed in 19 patients (6.1%). Recurrence occurred in 78 patients (24.8%) after treatment.

Among the 250 patients who initially underwent tube thoracostomy, 70 (28.0%) experienced a second pneumothorax, while 8 out of 45 patients (17.8%) who received oxygen therapy developed recurrence. No recurrence was observed in patients who underwent VATS.

### Outcomes after the Second Episode

Of the 78 patients who experienced a second pneumothorax, 35 (44.9%) were treated with VATS, 20 (25.6%) with tube thoracostomy, and 23 (29.5%) with oxygen therapy.

Among the 23 patients who received oxygen therapy for the second pneumothorax, 9 (39.0%) experienced a third episode, while 7 out of 20 (35.0%) who underwent tube thoracostomy and 3 out of 35 (8.6%) who underwent VATS developed a third episode (Table 1).

**Table 1:** Clinical and Demographic Characteristics

Age (years)^*^		28 (24-35)
Gender^#^	Male	292 (93.0)
	Female	22 (7.0)
Smoking^#^		232 (73.9)
Pneumothorax site^#^	Right	174 (55.4)
	Left	140 (44.6)
Treatment of First Pneumothorax^#^	Oxygen	45 (14.3)
	Tube Thoracostomy	250 (79.6)
	VATS	19 (6.1)
Treatment of Second Pneumothorax^#^	Oxygen	23 (29.5)
	Tube Thoracostomy	20 (25.6)
	VATS	35 (44.9)
Treatment of Third Pneumothorax^#^	Oxygen	9 (47.4)
	Tube Thoracostomy	7 (36.8)
	VATS	3 (15.8)

# n (%)

* Median (IQR 25-75)

### Comparison of Treatment Choice by Episode

Among the 45 patients who received oxygen therapy during the first pneumothorax episode, 8 (17.8%) experienced a second pneumothorax. Of these, 6 (75.0%) were treated with oxygen therapy and 2 (25.0%) with tube thoracostomy during the second episode.

**Figure 1:**
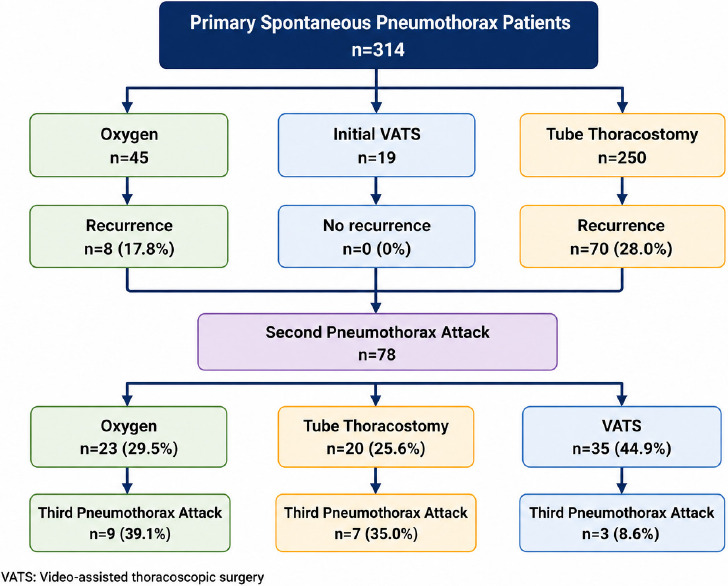
The Distribution of Recurrence Rates According to Treatment Methods Applied in the First, Second, and Third Episodes in Patients with Primary Spontaneous Pneumothorax

Among the 250 patients who underwent tube thoracostomy during the first episode, 70 (28.0%) experienced recurrence, and 35 (50.0%) of these were treated with VATS during the second episode. None of the 19 patients who underwent VATS during the first episode experienced a second or third pneumothorax (Figure 1).

### Relationship between Treatment Modality and Gender

No statistically significant difference was found between the treatment methods for pneumothorax episodes and gender (p>0.05 for all comparisons) (Table 2).

**Table 2:** Analysis of Variables by Gender

			Gender		Total	Fisher's Exact Test
			Female	Male		
First Pneumothorax	Oxygen	n (%)	1 (2.2)	44 (97.8)	45 (100)	p>0.05
	Tube Thoracostomy	n (%)	20 (8.0)	230 (92.0)	250 (100)	
	VATS	n (%)	1 (5.3)	18 (94.7)	19 (100)	
Total		n (%)	22 (7.0)	292 (93.0)	314 (100)	
Second Pneumothorax	Oxygen	n (%)	1 (4.3)	22 (95.7)	23 (100)	p>0.05
	Tube Thoracostomy	n (%)	0	20 (100)	20 (100)	
	VATS	n (%)	2 (5.7)	33 (94.3)	35 (100)	
Total		n (%)	3 (3.8)	75 (96.2)	78 (100)	
Third Pneumothorax	Oxygen	n (%)	1 (11.1)	8 (88.9)	9 (100)	p>0.05
	Tube Thoracostomy	n (%)	0	7 (100)	7 (100)	
	VATS	n (%)	0	3 (100)	3 (100)	
Total		n (%)	1 (5.3)	18 (94.7)	19 (100)	

### Relationship between Treatment Modality and Smoking Status

A statistically significant difference was found between the treatment methods for the first and second pneumothorax episodes and smoking status (p<0.001 and p=0.007). All patients who underwent VATS for both the first and second episodes were smokers (Table 3).

**Table 3:** Analysis of Variables by Smoking

			Smoking		Total	Fisher's Exact Test
			No	Yes		
First Pneumothorax	Oxygen	n (%)	19 (42.2)	26 (57.8)	45 (100)	***p<0.001***
	Tube Thoracostomy	n (%)	63 (25.2)	187 (74.8)	250 (100)	
	VATS	n (%)	0	19 (100)	19 (100)	
Total		n (%)	82 (26.1)	232 (73.9)	314 (100)	
Second Pneumothorax	Oxygen	n (%)	5 (21.7)	18 (78.3)	23 (100)	***p=0.007***
	Tube Thoracostomy	n (%)	2 (10.0)	18 (90.0)	20 (100)	
	VATS	n (%)	0	35 (100)	35 (100)	
Total		n (%)	7 (9.0)	71 (91.0)	78 (100)	
Third Pneumothorax	Oxygen	n (%)	1 (11.1)	8 (88.9)	9 (100)	p>0.05
	Tube Thoracostomy	n (%)	0	7 (100)	7 (100)	
	VATS	n (%)	0	3 (100)	3 (100)	
Total		n (%)	1 (4.3)	22 (95.7)	23 (100)	

## Discussion

The main finding of this study was that VATS performed during the first pneumothorax episode resulted in no recurrence, whereas recurrence was observed following both tube thoracostomy and oxygen therapy. This finding supports the growing body of evidence suggesting that VATS provides superior long-term outcomes in appropriately selected PSP patients^10^–^12^. The recurrence rate observed after tube thoracostomy in our series (28.0%) is consistent with previously reported values ranging from 16% to 52%^13^,^14^, whereas patients treated with oxygen therapy alone demonstrated increasing recurrence with each episode^13^–^15^. These results emphasize the risk of repeated pneumothorax in conservatively managed patients, particularly after the second episode.

In our study, 44.9% of patients with recurrent PSP underwent VATS at the second episode, and the recurrence rate markedly decreased thereafter. This supports findings from previous literature that surgical intervention plays a critical role in reducing recurrence, particularly after the second episode^16^,^17^. Various studies have reported recurrence rates of 4–11% after VATS^9^,^16^, consistent with our observation that none of the patients treated with VATS during the first episode experienced recurrence. Recent evidence also favors earlier surgical intervention; Pogorelić et al. demonstrated that VATS is successful, efficient, and safe, with significantly lower recurrence rates compared with chest tube insertion^12^. These observations suggest that earlier consideration of VATS in selected PSP patients may be justified^10^,^16^.

While surgery is not routinely recommended for all first-episode PSP patients, our findings support considering VATS in selected individuals. This approach may be particularly appropriate in patients with large pneumothoraces involving more than one-third of the hemithorax^3^, those in whom bullae or blebs are clearly identified on CT imaging suggesting structural susceptibility^8^, cases with persistent air leakage or failure of conservative management^13^, and high-risk occupational groups such as aircraft personnel and divers where recurrence poses significant danger^3^.

A notable finding of our study was the significant relationship between treatment modality and smoking status during both the first and second episodes. All patients who underwent VATS were smokers, suggesting that smoking may contribute to a more severe clinical course necessitating surgical intervention. Smoking is known to alter alveolar architecture, impair elasticity, and increase susceptibility to pneumothorax^18^,^19^. Our data are consistent with previous reports describing the adverse impact of smoking on PSP prognosis^6^,^18^. Therefore, smoking cessation should be emphasized as a critical component in the management of PSP^19^,^20^.

This study has limitations, including its retrospective, single-center design and non-randomized treatment selection, which may introduce selection bias. Smoking status was self-reported, and radiologic pneumothorax measurements were based on chest radiographs rather than CT in all cases. Additionally, long-term follow-up was not available for every patient. Larger prospective multicenter studies are needed to confirm these findings.

## Conclusions

This study demonstrated that VATS performed during the first pneumothorax episode resulted in no recurrence, whereas recurrence was commonly observed following tube thoracostomy and oxygen therapy. These findings indicate that early surgical intervention may provide superior long-term outcomes in appropriately selected PSP patients. The increasing recurrence rate with repeated conservative management suggests that prolonged non-operative treatment may not be optimal. Additionally, the significant association between smoking and treatment requirement highlights the importance of smoking cessation in the clinical management of PSP. Overall, our results support considering VATS earlier in the treatment algorithm for selected PSP patients, and further prospective studies are warranted to refine patient selection criteria and validate these findings.
